# Pro- and Anti-tumorigenic Effects of MSC Secretome in Glioblastoma: Mechanisms and Therapeutic Implications

**DOI:** 10.1007/s12015-026-11082-z

**Published:** 2026-02-14

**Authors:** Atiyeh Asadpour, Graeme S. Cottrell, Darius Widera

**Affiliations:** 1 Stem Cell Biology and Regenerative Medicine Group, School of Pharmacy, University of Reading, PO Box 226, Whiteknights, Reading, RG6 6AP UK; 2 Cellular and Molecular Neuroscience, School of Pharmacy, University of Reading, Reading, UK

**Keywords:** Mesenchymal stromal cells, Glioblastoma, Secretome, Extracellular vesicles, Potency assays

## Abstract

Despite advances in surgical resection, radiation, and chemotherapy, glioblastoma remains a lethal condition driven by intrinsic heterogeneity, therapy resistance, and an immunosuppressive tumour microenvironment. Mesenchymal stromal cells (MSCs) and their secretomes, comprising cytokines, growth factors, and extracellular vesicles, have emerged as promising therapeutic candidates due to their tumour-homing properties and anti-inflammatory and immunomodulatory potential. However, recent evidence reveals a paradox: MSC secretomes exhibit both anti-inflammatory/immunomodulatory potential and pro-tumorigenic activities, depending on MSC source, passage number, and environmental and manufacturing contexts. In this review, we critically examine the molecular mechanisms underlying these opposing effects, synthesising evidence on how MSC source, donor variability, passage number, and environmental priming/licensing (e.g., hypoxia, inflammatory licensing) dictate secretome composition and function. We identify critical manufacturing determinants, including the necessity for upper passage limits and standardised isolation protocols, and propose a translational framework that integrates mechanism-based potency assays, such as nuclear factor-κB (NF-κB) reporter systems and multi-donor mixed lymphocyte reactions, to predict clinical activity. Establishing these robust quality controls and mechanistic release and rejection criteria will be essential to resolve the functional plasticity of secretomes and enable the safe translation of MSC-based therapies for glioblastoma.

**Graphical Abstract:**

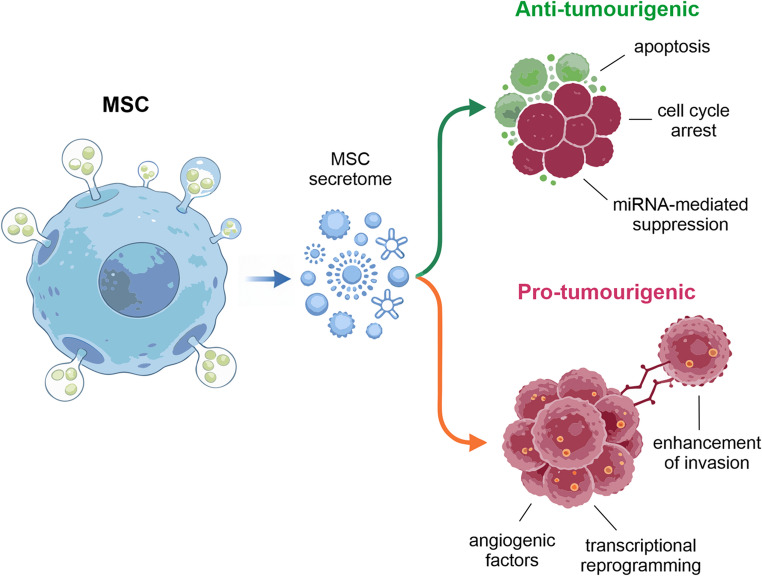

## Introduction

### Clinical Challenges and Biological Complexity of GBM

 The World Health Organisation classifies glioblastoma (GBM) as a grade IV astrocytoma, characterised by rapid proliferation, extensive infiltration into surrounding brain tissue, and vascular proliferation, contributing to its dismal prognosis [1]. Despite current multimodal therapy combining maximal surgical resection, radiation, and temozolomide chemotherapy, GBM is characterised by profound treatment resistance and near-universal recurrence. Patient outcomes remain devastating, with a median overall survival of only 15 months and a five-year survival rate below 5% [2, 3].

The poor prognosis of GBM reflects several interconnected biological challenges. Firstly, GBM exhibits aggressive infiltrative growth and high intra- and inter-tumoural heterogeneity, complicating complete surgical resection and uniform therapeutic responses [4, 5]. Secondly, intrinsic and acquired resistance mechanisms limit the efficacy of standard chemotherapy and radiotherapy [6]. Thirdly, GBM establishes a highly immunosuppressive tumour microenvironment (TME) that promotes tumour progression while suppressing anti-tumour immune responses [7, 8]. These challenges are further complicated by the presence of therapy-resistant glioma stem-like cells [9], extensive neovascularisation [10], and the capacity to recruit and reprogram stromal cells, collectively creating a self-sustaining tumorigenic niche [11, 12].

### Biology and MSCs and their Therapeutic Potential

**Fig. 1:**
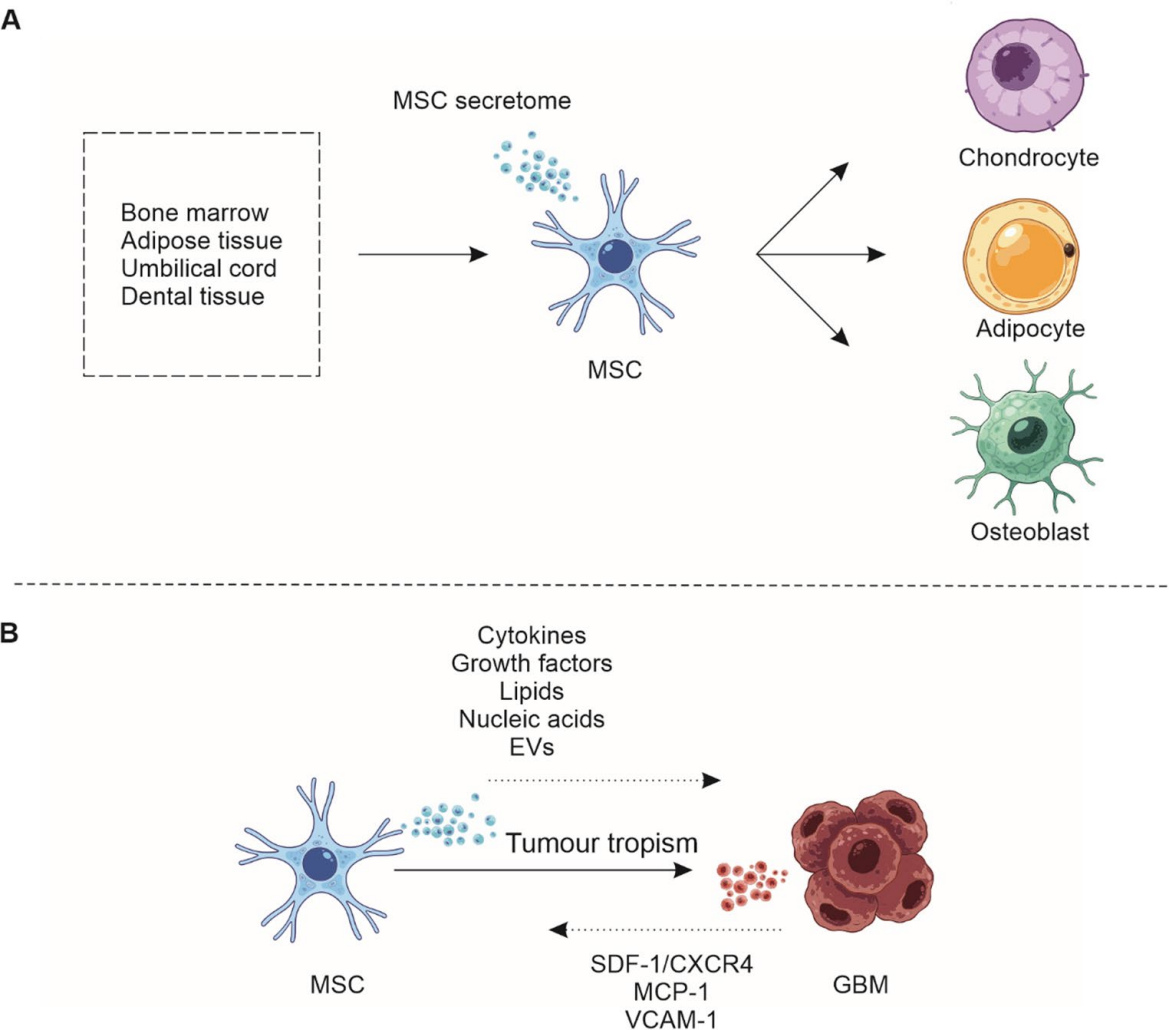
Biological characteristics and tumour-tropic mechanisms of mesenchymal stromal cells (MSCs). MSCs are multipotent progenitors derived from diverse sources (including bone marrow, adipose tissue, and umbilical cord) with the capacity for trilineage differentiation into adipocytes, chondrocytes, and osteoblasts. Beyond their regenerative potential, MSCs exhibit low immunogenicity and secrete a broad repertoire of bioactive immunomodulatory and reparative factors. Crucially, they demonstrate specific homing to the glioblastoma microenvironment driven by inflammatory and hypoxic gradients, mediated by key chemokines (e.g., stromal cell-derived factor-1 (SDF-1)/C-X-C chemokine receptor type 4 (CXCR4), monocyte chemoattractant protein-1 (MCP-1)) and adhesion molecules (e.g., vascular cell adhesion molecule-1 (VCAM-1)). GBM: glioblastoma, EVs: extracellular vesicles

MSCs are adult progenitor cells capable of differentiating into multiple mesenchymal lineages, including adipocytes, chondrocytes, and osteoblasts [13] (Fig. 1A).

MSCs can be readily isolated from diverse tissue sources, including bone marrow, adipose tissue, dental tissues, and umbilical cord [14]. MSCs exhibit low immunogenicity in their undifferentiated state due to absent or minimal expression of major histocompatibility complex class II and costimulatory molecules (e.g., CD40, CD80 and CD86), which facilitates allogeneic transplantation. However, inflammatory conditions and differentiation can upregulate major histocompatibility complex expression, leading to eventual immune recognition and clearance, typically within weeks to months of transplantation [15–17].

MSCs have become increasingly prevalent as therapeutics due to their accessibility from multiple tissue sources and relative ease of harvesting [18]. Importantly, tissue origin significantly influences both regenerative potential and therapeutic efficacy of MSCs [19, 20]. Since the first clinical trial in 1995, bone marrow-derived MSCs (BM-MSCs), umbilical cord-derived MSCs (UC-MSC), and adipose-derived MSCs (AD-MSCs have emerged as the primary clinical sources [21]. Beyond source-dependent effects, patient age, sex, extraction feasibility, and somatic mutation burden further affect the regenerative potential of MSCs [21]. In addition, non-standardised isolation techniques further complicate cross-study comparisons. To address this, Dominici et al. proposed standardised minimal criteria for the definition of MSCs. According to these guidelines, human MSCs must be plastic adherent, express CD105, CD73 and CD90, lack haematopoietic and endothelial lineage markers (CD45, CD34, CD14/CD11b, CD79α/CD19, HLADR) and be able to undergo trilineage differentiation in vitro (osteogenic, adipogenic, chondrogenic) [22]. Interestingly, despite their restriction to mesodermal differentiation, clinical trials have demonstrated the therapeutic efficacy of MSCs [23]. The broad therapeutic benefit is largely attributed to ‘bystander effects’, which involve MSC-driven paracrine modulation of endogenous repair mechanisms, primarily mediated by factors secreted in the MSC secretome [24]. Briefly, MSC secretomes exert their regenerative function through immunomodulation and reduced inflammation [25]. In addition, they support angiogenesis and facilitate endogenous regenerative processes through the secretion of paracrine factors [26, 27].

Importantly, MSCs possess inherent properties of migration toward damage, inflammation, hypoxia, and neoplastic growth, a phenomenon termed tumour tropism [28] (Fig. 1B). Tumour tropism is mediated by coordinated chemokine, cytokine, and adhesion molecule networks. Key mediators of this phenomenon include SDF-1)/CXCR4, MCP-1, and VCAM-1, which guide MSCs to pathological microenvironments [29].

These tumour-tropic properties and secretory capabilities of MSCs have positioned them as attractive cellular delivery platforms for targeted cancer therapeutics. In GBM, MSCs have been used pre-clinically as vectors to deliver immunomodulatory cytokines [30] and oncolytic viruses [31], benefiting from their ability to cross the blood-brain barrier and selectively home to tumour tissue. Secretomes offer a cell-free alternative to MSCs that mitigates key safety risks associated with whole cell transplantation, including ectopic differentiation and long-term engraftment. As non-viable, size filterable biologicals, secretomes can be manufactured under Good Manufacturing Practice (GMP) conditions with good batch-to-batch consistency and scalable production while avoiding immunogenicity concerns inherent to living cell products.

### MSC Secretomes

Secretomes produced by MSCs include diverse signalling molecules, which include cytokines, growth factors, chemokines, lipids, and nucleic acids [32]. Importantly, they are also rich in extracellular vesicles (EVs), which are membrane-bound particles released from cells that mediate intercellular communication [33]. EVs are heterogeneous in size, cargo composition, and biological function. The size differences enable classification of EVs into distinct subtypes, small EVs (sEVs) (< 200 nm) and large EVs (lEVs) (> 200 nm). EVs transfer diverse molecular cargo, including proteins, nucleic acids (mostly miRNAs and mRNAs), and lipids, between cells, thereby modulating both physiological and pathological processes in recipient cells. The nucleic acid content of EVs is of particular therapeutic interest, as the lipid bilayer protects enclosed RNA molecules from circulation-mediated degradation, enabling their delivery to target cells [34]. EVs also exert paracrine effects through surface ligand-receptor interactions that trigger downstream signalling cascades [35, 36]. EVs accomplish this through direct engagement of target cell receptors via surface-bound ligands and enzymes, thereby remodelling the extracellular microenvironment. This extracellular mode of action, referred to as `extracellular modulation by EV attributes`, enables efficient tissue repair and immunoregulation at relatively low EV concentrations.

Together, secretome constituents can modulate critical processes in cancer cells, such as angiogenesis, immune responses, cell survival, and matrix remodelling [37] .

The MSC secretome orchestrates microenvironmental remodelling through key regulatory factors, particularly vascular endothelial growth factor (VEGF), transforming growth factor-beta (TGF-β), hepatocyte growth factor (HGF), and IL-10, which collectively promote vascular regeneration, suppress inflammatory responses, and redirect immune cells toward pro-regenerative pathways [38]. However, in oncological contexts, the MSC secretome exhibits remarkable functional duality. A similar molecular repertoire can suppress tumour progression via anti-inflammatory and pro-apoptotic mechanisms, or paradoxically support tumour survival and angiogenesis, depending on the molecular composition modulated by environmental stressors [39]. This context-dependent plasticity underscores the critical need to understand microenvironmental determinants of the biological effects of secretomes when deploying MSC secretome-based therapeutics in cancer.

### Clinical Context and Need for this Review

The therapeutic potential of MSC secretomes in GBM is paradoxical. While certain MSC sources produce secretomes that suppress tumour progression, others promote invasion, proliferation, and survival. This functional duality highlights the need for a deep mechanistic understanding of the action of MSC-secretomes on GBM to enable the development of safe, predictable, source-optimised therapeutic strategies. This review addresses this knowledge gap via a comprehensive, integrated analysis of interactions of MSC-secretomes with GBM. 

### Effects of MSC Secretomes on GBM Cells

Recent research indicates that MSC secretomes possess potent anti-tumour properties through multiple distinct mechanisms, yet simultaneously can promote GBM progression under specific contexts. This section systematically examines both anti-tumorigenic and pro-tumorigenic pathways, providing a balanced mechanistic framework for understanding the functional duality of MSC secretomes in GBM biology.

## Anti-tumourigenic Mechanisms

### Induction of Apoptosis and Death Receptor Engagement

In 2014, Yang and colleagues demonstrated that secretomes from UC-MSCs have robust anti-glioma activity by inducing programmed cell death in GBM cells through caspase-dependent pathways [40]. In this study, the authors showed that both UC-MSC and AD-MSC conditioned media (CM) inhibited U251 glioma cell proliferation, with UC-MSC CM producing significantly more potent apoptotic effects compared to AD-MSC CM. The apoptotic mechanism involved upregulation of the pro-apoptotic caspases, caspase-3 and caspase-9, while anti-apoptotic proteins, including survivin and XIAP were dramatically downregulated. Building on these findings, Hardiany et al. reported that CM from UC-MSCs increases caspase-9 mRNA expression 1.6-fold in T98G GBM cells [41]. Notably, the secretome simultaneously upregulated death receptor 4 mRNA expression, establishing a cell surface death receptor competency for TRAIL-mediated extrinsic apoptosis (Table 1). Most strikingly, CM induced a strong upregulation of decoy receptor 1 mRNA expression.

**Table 1:** Mechanisms of MSC secretome-mediated modulation of GBM

Mechanism	MSC Source	Key Factors / Cargo	Biological Effect on GBM	Reference
Anti-Tumorigenic Effects				
Apoptosis induction	UC-MSCs (CM)	TRAIL; Upregulation of Caspase-3/9; Downregulation of XIAP/Survivin	Induced ~ 50% apoptosis in U251 cells; sensitised cells to cell death.	[40, 42]
Cell cycle arrest	UC-MSCs; WJ-MSCs (CM)	Secreted soluble factors (unidentified specific proteins)	Induced G0/G1 phase arrest (61–70% of cells); promoted glial differentiation (GFAP↑).	[40, 43]
miRNA-mediated suppression	BM-MSCs (engineered EVs)	miR-146b (via plasmid transfection)	Targeted EGFR and NF-κB; significantly reduced xenograft growth in vivo.	[44]
IGFBP-4 mediated inhibition	BM-MSCs (CM)	IGFBP-4	Sequestered IGF-1/2, blocking pAKT/ERK survival signalling; reduced neovascularisation.	[45]
*Pro-Tumorigenic Effects*				
Angiogenesis & chemotaxis	UC-MSCs; WJ-MSCs (CM)	VEGF, PDGF-BB, HGF, SDF-1	Promoted endothelial tube formation; enhanced GBM cell migration via CXCR4/c-Met.	[37, 46]
Invasion enhancement	BM-MSCs; AD-MSCs (co-culture/CM)	MMPs (MMP-2, -9), Cathepsin B, Calpain1	Context-dependent: Enhanced invasion of U373 cells (via proteases) but inhibited U87 invasion.	[47, 48]
Transcriptional reprogramming	AD-MSCs (CM)	Soluble factors inducing SOX4, H19	Shifted GBM cells toward a stem-like, more malignant transcriptional phenotype.	[49]
ECM remodelling	AD-MSCs; WJ-MSCs (CM)	Fibronectin, tissue inhibitor of metalloproteinase-1 (TIMP-1), LCN2	Increased tumour cell adhesion, motility, and proliferation via matrix modification.	[50]
Cell survival/anti-apoptosis	UC-MSCs	Survivin	Increased expression of Survivin	[41]
Angiogenesis/anti-apoptosis/survival	AD-MSCs	VEGF, angiopoietin 1, PDGF, ILGF, SDF-1/CXCL12	Increased angiogenesis and tumour growth	[42]
Expression of tumour stemness genes	AM-MSCs	SOX4, H19	Upregulation of SOX4 and H19	[49]
Angiogenesis and chemotaxis	UC-MSC	SDF-1, MCP1, HGF, VEGF and PDGFBB (a dimeric form of platelet-derived growth factor (PDGF)	Increased migration and angiogenesis	[46]
Increase of proliferation and migration	UC-MSC	CXCL1, CXCL2, CXCL3, and CXCL5	Activation of CXCR2	[51]
Maintenance of GSC stemness	Glioma-associated MSCs	IL-6	Activation of STAT3	[52]

The table summarises key anti-tumour and tumour-supportive effects of MSC-derived conditioned media and extracellular vesicles on GBM cells, organised by MSC source, principal secreted factors or cargo, and downstream biological effects. Entries are derived from preclinical in vitro, co-culture, and in vivo xenograft studies using human or rodent GBM models. CM: conditioned medium; evs: extracellular vesicles; UC: umbilical cord; BM: bone marrow; AD: adipose-derived; WJ: wharton’s jelly

While UC-MSC secretomes induced pro-apoptotic gene expression changes, anti-apoptotic survivin mRNA simultaneously increased, suggesting that UC-MSC secretomes induce a complex, partially reversible apoptotic priming state in GBM cells rather than irreversible cell death commitment (Table 1) [41]. This contextual limitation highlights the importance of complementary mechanisms to achieve sustained anti-tumour activity.

UC-MSC secretomes also contain TRAIL (tumour necrosis factor-related apoptosis-inducing ligand), a soluble death receptor ligand with potent pro-apoptotic activity. Akimoto et al. demonstrated that UC-MSCs secreted TRAIL at high levels. Using terminal deoxynucleotidyl transferase-mediated biotinylated UTP nick-end labelling (TUNEL) assays in a co-culture model, they determined that TRAIL promoted primary GBM cell apoptosis [42].

### Cell Cycle Arrest and Glial Differentiation

Beyond promoting apoptosis, MSC secretomes suppress GBM proliferation through cell cycle arrest mechanisms. In this context, it has been demonstrated that UC-MSC CM induced a G0/G1 cell cycle arrest in U251 cells in G0/G1 phase, preventing cell cycle progression through secreted factors [40]. Critically, this secretome-mediated cell cycle arrest was accompanied by enhanced expression of GFAP, a marker indicating differentiation toward a normal glial cell phenotype, with characteristic process outgrowth and morphological transformation to astrocytic morphology, all driven by secreted differentiation factors rather than cell-cell contact.

In 2021, Aslam et al. reported that Wharton’s jelly-derived MSC (WJ-MSC) secretomes and BM-MSC secretomes effectively inhibited U87 glioma cell proliferation and migration [43]. Cell cycle analysis revealed that secretome exposure induced G1 phase arrest as the primary mechanism, and morphological analysis showed neurite shortening and cytoplasmic vacuolation in treated GBM cells, indicating apoptotic and differentiation processes mediated by secreted bioactive components. WJ-MSC secretomes demonstrated particularly robust anti-tumour effects in serum-free conditions, highlighting that secreted factor bioactivity is independent of serum components and cellular context.

### miRNA-mediated Suppression of Oncogenic Pathways

EVs derived from MSCs transfer tumour-suppressive miRNAs to GBM cells, enabling anti-tumour effects independent of direct cell contact. Katakowski and colleagues demonstrated that sEVs isolated from BM-MSC secretomes engineered to express miR-146b, a miRNA with a central role in negative feedback control of inflammatory signalling, inhibited glioma growth both in vitro and in vivo, operating as cell-free delivery vehicles [44]. BM-MSC-derived sEVs encapsulated and transferred miR-146b to recipient glioma cells, where it reduced expression of epidermal growth factor receptor (EGFR) and NF-κB activity. Critically, a single intratumoral injection of purified miR-146b-containing MSC-derived sEVs significantly reduced glioma xenograft growth in rats, establishing proof-of-concept that cell-free sEVs mediate therapeutic anti-tumour effects without requiring intact MSCs.

Beyond engineered sEVs, unmodified MSC-derived EVs naturally transfer tumour-suppressor microRNAs. In particular, MSC secretomes contain sEVs carrying miR-124 and miR-145, which inhibit oncogenic pathways in GBM cells, repressing cell proliferation and increasing apoptosis [53]. A recent systematic review by Viktorsson et al. analysed preclinical studies exploring the effects of MSC-derived EVs on GBM [54]. Across the reviewed literature, MSC-EVs consistently attenuated glioma cell migration and invasiveness in vitro, largely through the delivery and upregulation of tumour-suppressive miRNAs. These EV-mediated effects included gene expression modulation, inhibition of invasive phenotypes, and suppression of pro-tumour signalling pathways. Overall, the review supports the therapeutic potential of MSC secretomes and EVs as cell-free approaches for limiting glioblastoma progression.

### Metabolic Interference and Growth Factor Sequestration

Furusaka and colleagues identified insulin-like growth factor binding protein-4 (IGFBP-4) as a critical anti-glioma component of BM-MSC secretomes [45]. This secreted IGFBP-4-mediated mechanism resulted in reduced phosphorylation of AKT, ERK, IGF-1 receptor beta, and p38 MAPK. Immunodepletion experiments specifically removing IGFBP-4 from BM-MSC-derived CM reversed the anti-tumour effects, confirming IGFBP-4 as the main secreted factor responsible for growth inhibition. Importantly, in vivo experiments demonstrated that BM-MSC secretome greatly inhibited both tumour growth and neovascularisation, reducing CD31^+^ blood vessel density.

Complementing these findings, Prateeksha and colleagues reported that secretomes from dental MSCs reduced both inflammation and proliferation of U87 glioblastoma cells through coordinated suppression of p38 MAPK and AKT and a simultaneous upregulation of reactive oxygen species [54]. Mechanistically, MSC secretome treatment downregulated pro-inflammatory markers while upregulating anti-inflammatory factors through reactive oxygen species [54].

## Pro-tumourigenic Mechanisms

Despite the strong body of evidence indicating anti-tumour effects, several studies have demonstrated that identical or nominally similar MSC secretomes can paradoxically promote GBM progression. This complexity highlights that identical MSC secretome preparations simultaneously contain both pro-tumorigenic factors and anti-tumorigenic factors, with the biological outcome determined by relative factor concentrations, microenvironmental signalling context, and recipient cell response pathways. The following sections systematically examine pro-tumorigenic pathways organised by functional category: angiogenesis, invasion, stem cell maintenance, and immune modulation.

### Angiogenesis and Vascular Promotion

Neovascularisation is a hallmark of GBM aggressiveness and a key source of oxygen and nutrients supporting tumour expansion. Several MSC sources secrete factors that robustly promote angiogenic processes. Shen and colleagues demonstrated that UC-MSC CM is pro-angiogenic and chemotactic and contains high levels of SDF-1, MCP1, HGF, VEGF and PDGFBB (a dimeric form of PDGF), which robustly stimulate endothelial migration and tube formation via CXCR4, CCR2 and cMet signalling [46]. Similarly, AD-MSC secretomes have been shown to be enriched in pro-angiogenic factors and promote GBM growth [42].

 In vivo studies substantiated this angiogenic potential. Administration of WJ-MSC CM in chorioallantois membrane models has been shown to enhance tumour mass formation and vascularisation significantly [37]. These effects have been attributed to an upregulation of classic angiogenic regulators, including VEGF, PDGF, and downstream Wnt signalling effectors. These findings reinforce the concept that factors secreted by MSCs can sustain tumour growth and promote supportive vascular networks essential for GBM progression [50, 55].

An important mechanism linking MSC secretomes to pro-angiogenic and pro-invasive effects involves IL-6-mediated STAT3 signalling. MSC-derived IL-6 acts both directly on GBM cells and indirectly on endothelial cells via JAK/STAT3, PI3K/AKT, and MAPK pathway activation, enhancing endothelial migration, angiogenesis, and GBM cell invasiveness [52].

Additionally, CXCR2 ligand-mediated signalling represents a mechanism by which pro-angiogenic and chemotactic factors promote GBM progression. Bajetto et al. demonstrated that UC-MSC secretomes contain both pro- and anti-tumorigenic factors whose relative abundance determines the net biological effect on GBM cells at given concentrations [51]. Paradoxically, UC-MSC secretomes promoted GBM cell proliferation and migration via CXCR2 pathway activation by secreted ligands CXCL1, CXCL2, CXCL3, and CXCL5. This complexity highlights that identical MSC secretome preparations simultaneously contain both pro-tumorigenic factors (CXCR2 ligands promoting migration) and anti-tumorigenic factors (caspase-activating molecules), with the biological outcome determined by relative factor concentrations, microenvironmental signalling context, and recipient cell response pathways.

### Invasion, Migration, and Extracellular Matrix Remodelling

MSC secretomes promote a more invasive and migratory phenotype through multiple mechanisms centred on extracellular matrix (ECM) remodelling and protease activation. Proteomic profiling of WJ-MSC and AD-MSC conditioned media identified several bioactive proteins directly involved in invasion processes, including fibronectin, TIMP-1, and lipocalin-2 (LCN2) [50, 56]. These molecules mediate ECM remodelling, tumour cell adhesion, and motility [57, 58]. Their interaction with GBM cells promotes cytoskeletal reorganisation, enhanced invasiveness, and acquisition of more aggressive phenotypes. Such paracrine signals can function as potent modulators of GBM, supporting tumour expansion within permissive stromal environments [59, 60].

Co-culture experiments further substantiated these findings. Breznik et al. demonstrated that the interaction between MSC and GBM cells in two- and three-dimensional culture systems promoted invasion and colony formation, along with elevated levels of integrin and matrix metalloproteinases (MMPs), particularly MMP-2 and MMP-9, which are crucial for ECM degradation [47]. Interestingly, BM-MSC and AD-MSC co-cultures enhanced invasion of certain GBM cell lines (e.g., U373 cells) via protease upregulation, while paradoxically inhibiting invasion in other lines (e.g., U87 cells), highlighting the importance of cell-line-specific and context-dependent responses.

Li et al. showed that BM-MSC CM paradoxically suppresses C6 glioma cell proliferation while simultaneously enhancing migratory and invasive capacity in vitro and driving a more infiltrative tumour phenotype in vivo [48]. This enhanced invasiveness was accompanied by upregulation of vimentin, sphingosine kinase-1 and other motility-associated proteins, a classic epithelial-to-mesenchymal transition (EMT) signature that renders GBM cells more capable of infiltration while potentially reducing proliferation.

### Glioma Stem-like Cell Maintenance and Therapeutic Resistance

A particularly important pro-tumorigenic mechanism involves the maintenance or enhancement of glioma stem-like cell (GSC) properties, which are associated with self-renewal capacity, therapy resistance, and long-term tumour maintenance. In U87 cells, AD-MSC CM induced a transcriptional shift toward malignancy, significantly increasing expression of the oncogenic and stemness-associated regulators SOX4 and H19, despite only modest changes in migration and apoptosis [49]. This transcriptional reprogramming represents a fundamental shift in the functional phenotype of GBM cells toward a more stem-like, less differentiated, and more chemoresistant state. The mechanisms underlying this stemness induction involve secreted soluble factors that activate stemness-associated transcriptional networks. The chemokine receptor CXCR4, which binds the MSC-secreted chemokine SDF-1 (CXCL12), plays a critical role in maintaining the glioma stem-like cell niche and conferring therapeutic resistance [61–63].

CXCR4 signalling via SDF-1 binding activates AKT and ERK pathways, promoting self-renewal and suppressing differentiation [61]. The paradox that the same SDF-1/CXCR4 axis promotes both angiogenesis (through endothelial cell activation) and stemness maintenance (through GSC niche support) highlights the multifunctional nature of MSC-derived factors and the context-dependent consequences of their secretion [64].

### Immune Suppression and Pro-tumourigenic Immune Evasion

MSC secretomes can create an immunosuppressive microenvironment that favours tumour progression through multiple mechanisms. Key anti-inflammatory factors including IL-10 and TGF-β, which, in appropriate contexts, exert immunomodulatory benefits, paradoxically support tumour growth when present in excess or within a permissive tumour microenvironment [65, 66]. These cytokines promote the expansion of regulatory T cells (Tregs), recruit myeloid-derived suppressor cells, and suppress anti-tumour immune responses [67, 68].

The pro-tumorigenic immune effects are particularly evident in the context of hypoxia-driven licensing and inflammatory priming. Hypoxic culture conditions, which reflect the GBM microenvironment, upregulate IL-6, IL-8, VEGF and HGF in MSC secretomes, factors that simultaneously promote both immune suppression and tumour angiogenesis through HIF-1α-dependent activation of STAT3 signalling and pro-angiogenic pathways [32, 69, 70]. While inflammatory licensing with cytokines such as IFNγ or TNF-α can enhance some immunomodulatory and anti-tumour properties through upregulation of immune checkpoint ligands and tryptophan-degrading enzymes, chronic or excessive IL-6 and IL-8 production drives a pro-tumorigenic Th17/myeloid-skewed immune response. This paradoxically supports GBM growth and resistance by promoting regulatory T cell conversion, recruiting myeloid-derived suppressor cells, and restricting cytotoxic T lymphocyte development [71–74].

Taken together, these data establish MSC secretomes as bifunctional modulators of GBM biology, capable of either constraining or amplifying malignant behaviour depending on their cellular source, priming conditions, and the prevailing tumour microenvironment. Anti-tumour effects typically emerge when pro-apoptotic, differentiation-inducing and miRNA-mediated pathways dominate, whereas enrichment in angiogenic, chemotactic, immunosuppressive or pro-invasive factors shifts the balance towards tumour promotion. Thus, any translational strategy based on MSC secretomes or EVs must therefore incorporate stringent source selection, standardised manufacturing and functional profiling, and ideally rational engineering to reinforce anti-tumour properties while minimising or eliminating pro-tumourigenic components.

## Critical Determinants of Secretome Activity in GBM and Implications for Clinical Translation

The biological activity of MSC secretomes is determined not only by MSC tissue source and tumour microenvironment, but also by manufacturing variables that critically influence composition, reproducibility and safety. For clinical translation, these variables must be tightly controlled, standardised and linked to mechanism-reflective potency assays.

A prerequisite for any secretome product is a well-defined cellular starting material. In addition to the initial minimal criteria for MSC definition [22], ISCT additionally recommended including immunopotency as a release criterion for advanced phase clinical trials involving MSCs [75]. However, these criteria, while necessary, are insufficient to ensure functional homogeneity and batch-to-batch consistency of secretomes. Beyond phenotype, donor‑to‑donor variability is a major determinant of secretome composition and function. Comparative analyses show that secreted protein profiles differ significantly between donors and are further modulated by tissue source and age, with older donors exhibiting reduced abundance of regenerative factors [76–78]. Dynamic metabolic labelling and secretome proteomics have demonstrated that both donor identity and passage numbers markedly influence soluble factor secretion. Importantly, no single passage window optimises proliferation and bioactive factor release across donors, highlighting the need to qualify each manufacturing batch rather than assuming interchangeability [79, 80]. Passage-to-passage changes are particularly relevant for GBM, as higher passage MSCs display reduced proliferative capacity, altered cytokine release, increased senescence and shifts in immunomodulatory output that can invert the balance between anti-tumour and pro-tumour activities. Thus, several groups and reviews recommend defining an upper passage limit in clinical-grade products and reporting passage number as a critical quality attribute for any secretome products [50, 79, 80]. In addition, the activity of MSC secretomes is highly sensitive to culture conditions, including oxygen tension, serum supplementation, two- and three-dimensional culture, and `licensing` stimuli [32, 81, 82]. Hypoxia, a process highly relevant to the GBM niche, upregulates IL-6, IL-8, VEGF and HGF in MSC secretomes [83], potentially influencing GBM proliferation, migration, angiogenesis, and immune suppression. Inflammatory licensing with cytokines such as IFNγ or TNF-α reshapes the secretome and EV cargo, enhancing some immunomodulatory and anti-tumour properties [84], but this could also increase variability between donors and sources. All these variables imply that nominally similar MSC populations can release secretomes with different, even opposing, effects on GBM, depending on preconditioning regime and duration.

In addition, process-related factors such as cell density at harvest, conditioning time, medium composition (xeno-free versus serum-containing), and freeze-thaw cycles can also affect EV yield, protein content and bioactivity [85–88]. For GBM-directed products, it is therefore essential to define a full process design space, including donor, tissue source, passage range (e.g., ≤P5), seeding density, and conditioning/licensing parameters, and to lock these into GMP-compliant standard operating procedures. Without this, the same product may oscillate between anti-tumour and tumour supportive profiles.

The wide range of methodologies used to isolate MSC secretomes further adds to this complexity. Depending on the specific study, the term MSC secretome can refer to whole CM (soluble proteins and EVs), EV-enriched fractions, and occasionally protein-only fractions, each generated by different isolation methods. Ultracentrifugation remains the most widely used technique to concentrate EVs, but it is time-consuming, can deform/damage vesicles and co-isolates protein aggregates and lipoproteins if not combined with density gradients or size exclusion chromatography [89–91]. Alternatives such as ultrafiltration, tangential flow filtration, size exclusion chromatography and precipitation (polyethene glycol or commercial kits) each have distinct biases in terms of yield, purity, co-isolated contaminants, and scalability. Comparative studies show that the isolation method significantly alters EV cargo and downstream bioactivity, meaning that results obtained with crude CM, ultracentrifugation-derived EVs or precipitated EVs cannot be directly extrapolated to each other [92]. To address this, the International Society for Extracellular Vesicles (ISEV) released the Minimal Information for Studies of Extracellular Vesicles 2023 (MISEV2023) guidelines, establishing a consensus framework for EV nomenclature, pre-analytical variables, separation, and characterisation. Briefly, ISEV recommend transparent reporting of source, starting volume and all isolation steps, multiparametric characterisation, including particle size and concentration, reporting on EV-enriched markers (e.g., CD9, CD63, CD81) and negative markers for non-EV contaminants; and functional studies with appropriate controls to discriminate EV-mediated from soluble factor effects. For GBM-directed secretome products, adherence to MISEV2023 will be crucial to ensure that the mechanistic claims are reproducible and not confounded by protein or lipoprotein contaminants.

A major bottleneck for clinical translation of secretome-based GBM therapies is the lack of robust, mechanism-linked potency assays for MSC secretome products. Regulatory guidance for biologicals and cell therapies requires that potency assays quantitatively reflect the intended mechanism of action (MoA) and predict clinical activity. However, most current secretome/GBM studies rely on descriptive in vitro readouts such as viability or migration that are difficult to standardise across centres. Recent frameworks propose adapting established principles for cell‑based products to secretome‑based therapeutics by defining primary MoA-linked endpoints, identifying surrogate biochemical markers that correlate with these functional effects, and using orthogonal assays such as transcriptomic signatures to confirm pathway engagement [93]. For GBM, potency assays could assess endpoints such as caspase‑3/9 activation, STAT3 inhibition, reduction in GBM stemness markers, suppression of IL‑8 signalling, and surrogate biochemical markers such as IGFBP‑4 concentration for anti‑IGF signalling. As tumour-promoting inflammation is one of the key hallmarks of GBM, we propose addressing this by the implementation of NF-κB-pathway-specific reporter assays that provide quantitative, standardised readouts.

**Fig. 2:**
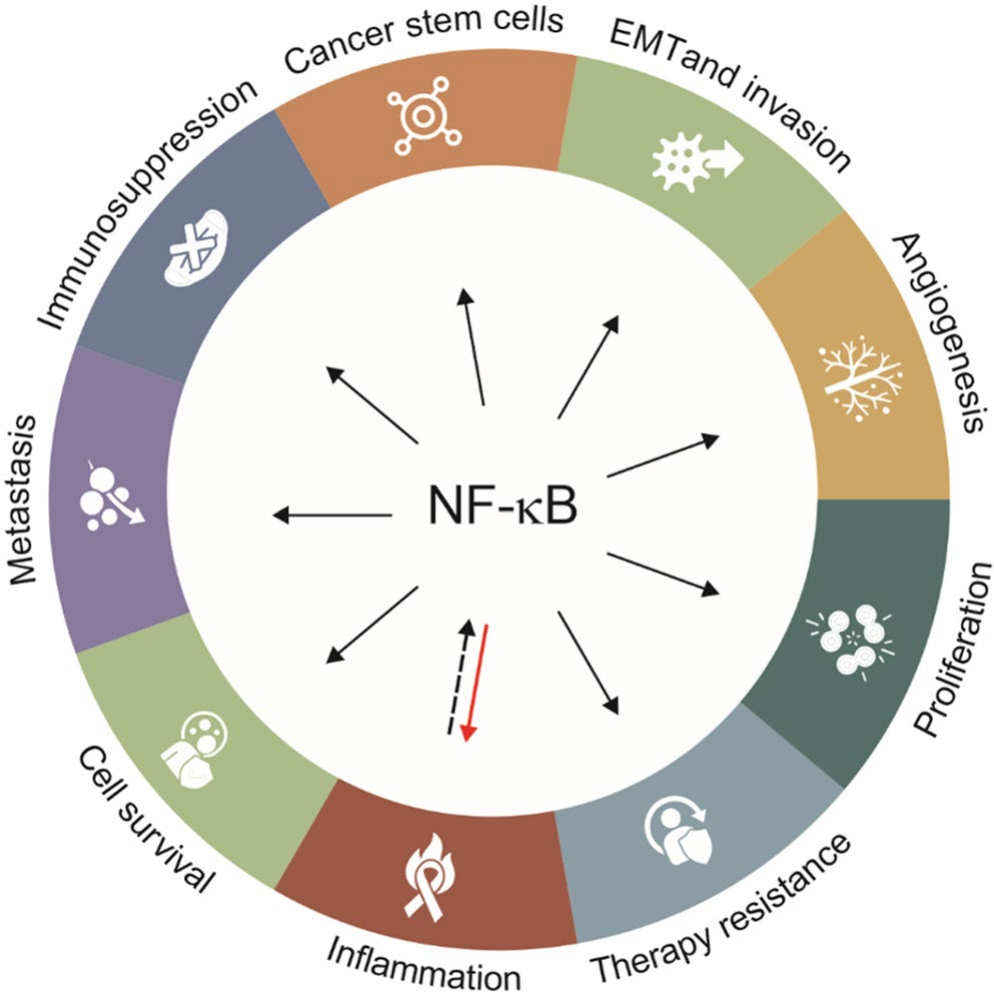
NF-κB serves as a master transcriptional hub linking inflammatory signals to multiple hallmarks of GBM. Constitutive activation of the NF-κB pathway drives diverse pro-tumorigenic processes, including cell survival, proliferation, angiogenesis, therapy resistance, and metastatic dissemination. It also orchestrates critical microenvironmental interactions by promoting immunosuppression, inflammation (via cytokines such as TNF-α, IL-1β, and IL-8), and the maintenance of cancer stem cell populations. Additionally, NF-κB signalling supports epithelial-to-mesenchymal transition (EMT) and invasion, further fuelling tumour aggressiveness. This broad regulatory influence positions NF-κB as a pivotal mechanistic node in GBM biology and a rational target for secretome-based potency assays to predict the therapeutic impact of MSC-derived products. Adapted from [99]

NF-κB acts as a critical link between inflammation and tumour progression by regulating the expression of pro-inflammatory cytokines, survival factors, and oncogenes [94]. In GBM, its constitutive activation creates a pro-tumorigenic microenvironment that sustains stem-like properties, increases migration and angiogenesis, and mediates resistance to standard radio- and chemotherapy (Fig. 2) [95–98].

NF-κB reporter GBM cells, as described in [100], would offer a high-throughput platform to measure secretome-mediated modulation of this central inflammatory and survival pathway.

An attractive complementary strategy to capture immunomodulatory potency is the multi-donor mixed lymphocyte reaction assay [101]. In this system, pooled allogeneic peripheral blood mononuclear cells are co-cultured with MSC-derived EVs, and modulation of T cell activation (CD25/CD54 on CD4⁺/CD8⁺ T cells) and monocyte subset distribution is quantified as a functional readout of EV-mediated immune suppression. As the assay is standardised, reproducible, and directly reports on key adaptive immune endpoints, it could be adapted to evaluate whether GBM-directed MSC secretomes or EV fractions exert the desired immunoregulatory profile while avoiding excessive immune suppression.

Regardless of the exact nature of the assays, potency-assay design must account for passage-to-passage and donor variability. Multi-parameter assay matrices have been developed for MSCs that integrate immunosuppression, trophic factor secretion and viability to classify lots as high or low potency [102]. Analogous matrices tailored to GBM, combining, for example, NF-κB activity, immunopotency, effects on glioma stem-like cells sphere formation, invasion through Matrigel^®^ and resistance to temozolomide, will be needed for secretome-based products. Embedding such assays early in process development will allow definition of acceptable potency windows and rejection of batches that drift toward pro-tumorigenic profiles. In GBM, where MSC secretomes can be either strongly anti- or pro-tumour, these determinants will require particular attention.

Consequently, in addition to release criteria, rejection criteria must be operationally defined through appropriate assays. Suitable rejection criteria should include increased proportion of glioma stem-like cells (assessed by neurosphere formation assays), elevated angiogenic potential (evaluated by endothelial migration and tube formation assays), and high levels of immunosuppressive cytokines such as IL-10 and TGF-β (quantified by multiplex cytokine assays). These negative selection criteria ensure that only batches with robust anti-tumour and immunoregulatory properties advance to clinical use.

Minimal MSC criteria ensure a baseline definition of the producing cell, but do not constrain secretome function, which is shaped by donor, passage, inflammatory licensing, hypoxia and manufacturing details. Similarly, EV-specific standards (MISEV2023) provide a rigorous framework for isolating and characterising vesicular fractions, but must be integrated with GBM-relevant potency assays and clearly defined clinical MoA to avoid deploying products with unintended tumour-supportive activities.

For GBM-directed MSC secretome therapies, a credible translational pathway therefore requires MSC products that meet ISCT minimal and updated safety criteria. This must include tightly controlled donor selection, passage limits and culture conditions, EV/secretome isolation and characterisation aligned with ISEV/MISEV recommendations, and validated, MoA-linked potency assays as formal release tests. Only by embedding these determinants into GMP-compliant manufacturing and trial design can MSC secretome-based interventions be advanced to the clinic with a reasonable expectation of consistent anti-tumour activity and minimised risk of inadvertently promoting GBM progression.

## Concluding Remarks

MSC secretomes offer a promising, cell-free approach to target GBM heterogeneity and immune evasion, yet their dual nature, capable of both suppressing and promoting tumour progression, demands rigorous translational control. To exploit their therapeutic potential while ensuring safety, future clinical strategies must prioritise two key pillars.

Firstly, standardisation of the biological source is critical. As donor identity, tissue origin, and passage number profoundly dictate secretome composition, clinical-grade manufacturing must implement strict donor selection criteria and define upper passage limits to prevent senescence-associated pro-tumorigenic shifts.

Secondly, mechanism-based potency testing must replace descriptive readouts. Integrating validated assays such as NF-κB reporter systems and multi-donor mixed lymphocyte reaction assays into lot release testing will ensure that every batch delivers the intended immunomodulatory and anti-tumour activity, filtering out subpotent or deleterious preparations and batches with pro-tumour activity.

## Data Availability

No datasets were generated or analysed during the current study.
